# Measurements of body fat distribution: assessment of collinearity with body mass, adiposity and height in female adolescents

**DOI:** 10.1016/j.rpped.2014.11.011

**Published:** 2015-03

**Authors:** Patrícia Feliciano Pereira, Hiara Miguel Stanciola Serrano, Gisele Queiroz Carvalho, Sônia Machado Rocha Ribeiro, Maria do Carmo Gouveia Peluzio, Sylvia do Carmo Castro Franceschini, Silvia Eloiza Priore

**Affiliations:** Universidade Federal de Viçosa, Viçosa, MG, Brazil

**Keywords:** Obesity, Adolescents, Body fat distribution, Anthropometry

## Abstract

**OBJECTIVE:**

: To verify the correlation between body fat location measurements with the body mass index (BMI), body fat percentage (BF%) and height, according to the nutritional status in female adolescents.

**METHODS:**

: A controlled cross-sectional study was carried out with 113 adolescents (G1: 38 with normal weight, but with high body fat level, G2: 40 with normal weight and G3: 35 overweight) from public schools in Viçosa-MG, Brazil. The following measures were assessed: weight, height, waist circumference (WC), umbilical circumference (UC), hip circumference (HC), thigh circumference, waist-to-hip ratio (WHR), waist-to-height ratio (WHtR), waist-to-thigh ratio (WTR), conicity index (CI), sagittal abdominal diameter (SAD), coronal diameter (CD), central (CS) and peripheral skinfolds (PS). The BF% was assessed by tetrapolar electric bioimpedance.

**RESULTS:**

: The increase in central fat, represented by WC, UC, WHtR, SAD, CD and CS, and the increase in peripheral fat indicated by HC and thigh circumference were proportional to the increase in BMI and BF%. WC and especially the UC showed the strongest correlations with adiposity. Weak correlation between WHR, WTR, CI and CS/PS with adiposity were observed. The height showed correlation with almost all the fat location measures, being fair or weak with waist measurements.

**CONCLUSIONS:**

: The results indicate colinearity between body mass and total adiposity with central and peripheral adipose tissue. We recommend the use of UC for assessing nutritional status of adolescents, as it showed the highest capacity to predict adiposity in each group, and also showed fair or weak correlation with height.

## Introduction

Adolescence starts with the bodily changes of puberty, being a period of major psychosocial and physical changes. Among these, it is worth mentioning the intense growth that interferes with the accumulation and distribution of body fat.^1^^,^ 2 Clinical and epidemiological studies have established that body fat distribution is related to cardiovascular risk factors in adults^3^^,^ 4 and also in children and adolescents.^5^^,^ 6 The use of valid measures when assessing body composition and the fat distribution pattern is required in population studies and clinical practice to attain an early identification of individuals at risk of developing diseases, and to help in the prevention/treatment of obesity.^7^

Body fat distribution can be assessed by different methods, such as computed tomography (CT) and magnetic resonance imaging (MRI), equipment which are more precise and directly measure the amount of visceral fat; however, they are high-cost methods that require extensive training of evaluators, and additionally, CT involves radiation exposure.^7^ Dual-energy X-ray absorptiometry (DXA) - as well as anthropometry and bioelectrical impedance analysis (BIA) - does not differentiate between subcutaneous and visceral fat. BIA, although not the most accurate method for assessing body composition, is a fast and convenient method for use in field studies.^8^^,^ 9 Anthropometric measurements include body circumferences, skinfold thickness and some diameters, which have the advantage of being relatively simple, inexpensive and non-invasive, and have a good performance in the prediction of visceral fat and cardiovascular risk.^10^^,^ 11

Several anthropometric measurements of body fat have been used in children and adolescents, although the best measure for the pediatric population is yet to be defined.^2^^,^ 5 ^,^ 6 ^,^ 12 It is unclear whether the increase in adiposity in children and adolescents is related to the increase in intra-abdominal fat.^13^ Thus, the present study aimed to investigate the correlation between peripheral and central fat measurements proposed in the literature with BMI, body fat percentage and height, according to the nutritional status of adolescent girls. 

## Methods

We performed a cross-sectional study with 113 female adolescents, aged 14 to 19 years, from public schools in the city of Viçosa - MG. A screening was carried out in schools to select the participants, using the measures of height and weight to determine BMI, as well as measures of body fat percentage (BF %) by BIA (Tanita^(r)^, Model 2220, Illinois, USA). The adolescents were also asked whether they had had menarche, and its date of occurrence. Measurements were obtained individually in a room or area established for that purpose inside the schools. Adolescents that met the criteria were invited for a second evaluation carried out by the Section of Nutrition of the Division of Health of Universidade Federal de Viçosa (UFV), where anthropometric and body composition measures were collected. The final sample consisted of 38 normal weight adolescents (BMI percentile between 5 and 85)^14^ but with high body fat percentage (>28%) (G1-Study group), 40 adolescents with normal weight according to BMI and normal fat percentage (20-25%) (G2-control group), and 35 with overweight risk/overweight classified according to the Center for Disease Control and Prevention (CDC) curves (BMI percentile ≥85)^14^ and high body fat percentage (>28 %) (G3-control group). The teenagers included in the study reported the occurrence of menarche for at least 1 year, which corresponds to a greater chance of having overcome the most intense period of physical transformations inherent to puberty.^15^ Sample size calculation was carried out with Epi Info 6.04 (CDC, Epi Info(tm) 6, Atlanta, USA) for cross-sectional studies, considering a population of the municipality of 4,507 individuals^16^ in the age range and gender of the study, prevalence of excess body fat estimated at 25%,^15^ 10% variability and 95% confidence interval, resulting in a minimum sample size of 35 subjects for each group. 

This study was approved by the Research Ethics Committee for Human Subjects at UFV. Participation was voluntary after verbal explanation, and after the free and informed consent form was signed by the adolescents and their parents and/or guardians.

Weight was measured in an electronic digital scale with a capacity of 150 kg and precision of 50g. Height was measured using a stadiometer with a length of 2.00m, divided into centimeters and subdivided in millimeters. All measurements followed the techniques proposed by Callaway.^17^ The BMI was calculated as the ratio between total body weight (kg) and height (m^2^).

The percentage of body fat was assessed by tetrapolar electrical bioimpedance analysis (Biodynamics^(c)^, model 310, version 7.1, Washington, USA). The assessment was carried out between 7:00 am and 8:30 am, after a 12-hour fasting and following the specific protocol for this type of evaluation.^18^

Waist circumference was measured at two locations: smallest abdominal circumference (waist circumference) and at the umbilicus (umbilical circumference), under the clothes and at the end of a normal expiration, using a flexible and inelastic measuring tape.^17^ The hip was measured at the greatest circumference of the gluteal region,^17^ over light clothing. Thigh circumference was measured 3 cm above the patella on the left side of the body in individuals whose right hand was dominant, and on the right side of the body in those whose left hand was dominant.^19^ Measurements were taken twice, and the mean value was used in the analysis. Waist-to-hip ratio (WHR) was calculated using the waist circumference and hip circumference measures; waist-to-thigh ratio (WTR) by dividing the umbilical circumference by thigh circumference, and waist-to-height ratio (WHtR) through the ratio between waist circumference and height. 

The conicity index (CI) was calculated through the following formula:^12^


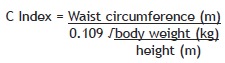


The distance between the back and the abdomen (sagittal abdominal diameter, SAD), and the distance between the iliac crests (coronal diameter, CD) were measured with the adolescents in the supine position, knees bent on a flat, firm surface, under the clothes and after a normal expiration. The midpoint between the iliac crests was identified and then the reading was performed at the level of the right iliac crest, taking care not to compress the tissues, using a metal caliper with an extension of 50cm, divided into centimeters and subdivided into millimeters (Cescorf^(r)^, Rio Grande do Sul, Brazil).^11^^,^ 20

Subscapular, suprailiac, triceps and biceps skinfolds were assessed on the right side of the body, and all measurements were taken by a single evaluator. Each measurement was verified three times, non-consecutively (using the mean value), with a Lange Skinfold Caliper.^21^ The measurement was repeated in case of divergence >10% between the three values. Peripheral skinfold (PSF) consisted of the sum of triceps and biceps folds, and central skinfold (CSF), of the sum of the subscapular and suprailiac folds, from which we calculated the CSF/PSF ratio.^22^

For the statistical analysis, the distribution of variables was verified through the Kolmogorov-Smirnov test. Exploratory analysis of data was carried out by measures of central tendency and dispersion. Subsequently, the Mann-Whitney test and/or Student's *t* test were used to identify statistical differences in study variables between the three groups of nutritional status, according to variable distribution. Pearson's and Spearman's correlation was performed between anthropometric variables and body composition with measures of fat distribution, according to the normality of the variables. The qualitative assessment of the degree of correlation between the variables followed the Callegari-Jacques criteria^23^ (null correlation: r=0; weak: 0-0.3; fair: 0.3-0.6, strong: 0.6-0.9, very strong: 0.9-1). Analyses were performed using Sigma-statistic 2.0 and STATA software, version 11.0 (StataCorp LP, Texas, USA). The statistical significance was set at *p*<0.05. 

## Results

The characteristics of the study population are shown in Table 1. Mean age and height did not differ between the groups, reflecting homogeneity between them. The variables weight, BMI, BF%, waist circumference (WC), umbilical circumference (UC), hip, thigh, WHtR, SAD, CD, WTR and PSF in group G1 were significantly higher than in G2, and lower than in G3 (*p*<0.001). WHR and WTR did not differ between G1 and G2. No statistically significant differences were observed between G1 and G3 regarding WTR, CI, and CSF/PSF ratio. 

**Table 1:** Age, anthropometry and body composition of the adolescents.

	Group 1 (n=38)		Group 2 (n=40)		Group 3 (n=35)	
	Mean ± SD	Median (range)	Mean ± SD	Median (range)	Mean ± SD	Median (range)
Age (years)	15.9 (1.3)	15.6 (14-18.8)	15.9 (1.3)	15.8 (14-18)	15.7 (1.1)	15.4 (14-17.9)
Weight (kg)	57.7 (6.3)	57. (46.5-75.4)	51.2 (6.0)^a.d^	49.9 (43-67.8)	70. (12.7)	67.5 (55.4-116)^b.d^
Height (m)	1.62 (0.06)	1.62 (1.50-1.80)	1.61 (0.07)	1.61 (1.48-1.78)	1.6 (0.06)	1.59 (1.49-1.74)
BMI (kg/m^2^)	21.9 (1.75)	21.7 (19.2-25.2)	19.7 (1.5)	19.4 (17.8-23.4)^a.d^	27.3 (4.03)	25.9 (23.4-41.4)^b.d^
BF%	30.6 (1.8)	30.2 (28.2-35.0)	22.7 (1.3)	22.9 (20.1-24.7)^a.d^	33.6 (3.3)	32.6 (29.2-42.4)^b.d^
WC (cm)	70.9 (7.9)	69.6 (61.2-111.0)	65.2 (3.1)	64.7 (60.4-72.7)^a.d^	79.1 (7.8)	76.8 (67.6-105.2)^b.d^
UmC (cm)	79.1 (5.5)	78.9 (69.0-91.9)	72.3 (4.3)	72.3 (64.8-83.7)^a.d^	89.5 (9.4)	88.0 (73.8-88)^b.d^
HC (cm)	97.8 (5.3)	97.2 (89.0-109.6)	91.8 (4.8)^a.d^	91. (86-104.7)	106.7 (8.0)^b.d^	105.3 (94.9-134.8)
Thigh (cm)	40.4 (2.7)	40.7 (34.5-46)	37.9 (2.3)^a.d^	37.6 (34.5-43)	44.8 (4.1)	44.4 (38.5-57.9)^b.d^
WHR	0.73 (0.09)	0.72 (0.62-1.21)	0.71 (0.03)	0.71 (0.6-0.8)	0.74 (0.05)	0.74 (0.66-0.85)^b.c^
WHtR	0.44 (0.05)	0.43 (0.37-0.72)	0.41 (0.02)	0.41 (0.36-0.45)^a.d^	0.50 (0.05)	0.48 (0.43-0.63)^b.d^
WTR	1.96 (0.13)	1.95 (1.73-2.25)	1.91 (0.11)	1.91 (1.7-2.1)	2.00 (0.13)	2.00 (1.70-2.30)
CI	1.07 (0.12)	1.06 (0.90-1.70)	1.04 (0.03)^a.c^	1.04 (0.9-1.1)	1.08 (0.04)	1.09 (0.90-1.20)
SAD	17.4 (0.9)	17.3 (15.3-18.9)	15.9 (1.1)^a.d^	15.8 (14.2-18.2)	20. (2.3)	19.7 (16.5-26.3)^b.d^
CD	30. (1.7)	30.2 (26.5-32.9)	27.7 (1.6)^a.d^	27.5 (25-31)	33.4 (2.7)^b.d^	33.8 (28.5-39.2)
CSF	56.5 (12.8)	53.5 (32.0-77.0)	39.2 (7.6)	38.0 (20.0-69.0)^a.d^	72.9 (13.5)^b.d^	72.0 (55.0-114.0)
PSF	38.5 (6.5)	38.5 (27.0-52.0)	30.8 (5.4)	30.0 (23.0-49.0)^a.d^	49.9 (9.9)^b.d^	49.0 (33.0-72.0)
CSF/PSF	1.47 (0.26)	1.43 (1.00-2.29)	1.28 (0.19)^a.d^	1.29 (0.69-1.57)	1.48 (0.21)	1.50 (1.08-1.78)

BMI, body mass index; BF%, body fat percentage; WC, waist circumference; UmC, umbilical circumference; HC, hip circumference; WHR, waist/hip ratio; WHtR, waist/height ratio; WTR, waist/thigh ratio; CI, conicity index; SAD, sagittal abdominal diameter; CD, coronal diameter; CSF, central skinfolds; PSF, peripheral skinfolds; CSF/PSF, central/peripheral skin folds. Student's t Test and Mann-Whitney Test. ^a^ Difference between G1 and G2. ^b^ Difference between G1 and G3. ^c^ p<0.05. ^d^ p<0.001.

Table 2 shows the correlation coefficient between the anthropometric and body composition variables in the total population. BMI and BF% were strongly correlated with measures of distribution of body fat, except WHR, WTR, CI and CSF/PSF. For these, the correlations were weak to fair. The strongest correlations were found between BMI and WC (r = 0.90, *p*<0.001) and between BF% and UC (r=0.76, *p*<0.001). Height showed a positive statistically significant correlation (although weak) with HC and a negative one with WHtR.

**Table 2:** Coefficient of correlation between measures of fat distribution with total body mass, body fat percentage and height in the total population (n=113).

Variables	Measures of fat distribution												
	WC	UmC	HC	Thigh	WHR	WHtR	WTR	CI	SAD	CD	CSF	PSF	CSF/PSF
BMI	0.90^b.d^	0.89^b.d^	0.89^b.d^	0.88^b.d^	0.28^b.c^	0.89^b.c^	0.25^b.c^	0.34^b.d^	0.86^b.d^	0.83^b.d^	0.82^b.d^	0.77^b.d^	0.41^b.d^
BF%	0.73^b.d^	0.76^b.d^	0.72^b.d^	0.67^b.d^	0.26^b.c^	0.71^b.c^	0.31^b.d^	0.39^b.d^	0.74^b.d^	0.67^b.d^	0.75^b.d^	0.69^b.d^	0.37^b.d^
Height	0.14^b^	0.18^b^	0.26^a.c^	0.14^a^	—0.13^a^	—0.26^b.c^	0.09^a^	—0.04^a^	0.11^b^	0.14^b^	—0.009^b^	—0.02^b^	0.01^a^

BMI, body mass index; BF%, body fat percentage; WC, waist circumference; UmC, umbilical circumference; HC, hip circumference; WHR, waist/hip ratio; WHtR, waist/height ratio; WTR, waist/thigh ratio; CI, conicity index; SAD, sagittal abdominal diameter; CD, coronal diameter; CSF, central skinfolds; PSF, peripheral skinfolds; CSF/PSF, central/peripheral skin folds. ^a^ Pearson's correlation test. ^b^ Spearman's correlation test. ^c^ p<0.01. ^d^ p<0.001.

Table 3 shows the correlation coefficient in the group of adolescents with normal weight but with excess body fat. BMI showed a significant correlation with WC, UC, HC, WHtR, SAD, CD, CSF and PSF. The BF% was not correlated with any measure of fat distribution, whereas height was correlated, but at a fair degree, with HC and WHtR. 

**Table 3:** Coefficient of correlation between measures of fat distribution with total body mass, body fat percentage and height in normal weight adolescents with high body fat (G1) (n=38).

Variables	Measures of fat distribution												
	WC	UmC	HC	Thigh	WHR	WHtR	WTR	CI	SAD	CD	CSF	PSF	CSF/PSF
BMI	0.69^b,e^	0.63^b,e^	0.78^b,e^	0.78^b,e^	0.07^b^	0.69^b,e^	—0.05^b^	0.09^b^	0.51^b,d^	0.59^b,e^	0.51^b,d^	0.61^b,e^	0.19^b^
BF%	0.26^b^	0.16^b^	0.05^b^	0.12^b^	0.16^b^	0.26^b^	0.06^b^	0.26^b^	0.14^b^	—0.15^b^	0.12^b^	0.17^b^	—0.03^b^
Height	0.06^b^	0.25^a^	0.40^a,c^	0.31^a^	—0.15^b^	—0.45^b,d^	—0.07^a^	—0.08^b^	0.10^a^	0.17^a^	0.07^a^	—0.02^a^	0.10^a^

BMI, body mass index; BF%, body fat percentage; WC, waist circumference; UmC, umbilical circumference; HC, hip circumference; WHR, waist/hip ratio; WHtR, waist/height ratio; WTR, waist/thigh ratio; CI, conicity index; SAD, sagittal abdominal diameter; CD, coronal diameter; CSF, central skinfolds; PSF, peripheral skinfolds; CSF/PSF, central/peripheral skin folds. ^a^ Pearson's correlation test. ^b^ Spearman's correlation test. ^c^ p<0.05. ^d^ p<0.01. ^e^ p<0.001.

In adolescents with normal weight and adequate body fat content, BMI showed a statistically significant fair to strong correlation with virtually all measures of fat distribution, except WHR, WTR, IC and PSF. The BF% showed a fair correlation with WC, UC, HC, thigh and SAD. As for the measures of fat distribution and height, they showed a fair correlation with WC, UC, HC, SAD, CD and WHtR. The correlation with WHtR was a negative one (r=-0.44) (Table 4).

**Table 4:** Coefficient of correlation between measures of fat distribution with total body mass, body fat percentage and height in adolescents with normal weight (G2) (n = 40).

Variables	Measures of fat distribution												
	WC	UmC	HC	Thigh	WHR	WHtR	WTR	CI	SAD	CD	CSF	PSF	CSF/PSF
BMI	0.68^b^^‡^	0.67^b^^‡^	0.58^b‡^	0.78^b‡^	—0.02^b^	0.73^b‡^	—0.13^b^	—0.03^b^	0.58^b‡^	0.49^b†^	0.33^b.c^	0.06^b^	0.34^b.c^
BF%	0.42^b.d^	0.43^b.d^	0.42^b.d^	0.31^b.c^	—0.19^b^	—0.03^b^	0.05^b^	—0.01^b^	0.46^b.d^	0.34^b^	0.18^b^	0.02^b^	0.16^b^
Height	0.45^a.d^	0.50^a.d^	0.55^a.e^	0.27^a^	—0.19^a^	—0.44^a.d^	0.22^a^	—0.03^a^	0.36^a.c^	0.44^a.c^	0.06^a^	—0.02^b^	0.14^a^

BMI, body mass index; BF%, body fat percentage; WC, waist circumference; UmC, umbilical circumference; HC, hip circumference; WHR, waist/hip ratio; WHtR, waist/height ratio; WTR, waist/thigh ratio; CI, conicity index; SAD, sagittal abdominal diameter; CD, coronal diameter; CSF, central skinfolds; PSF, peripheral skinfolds; CSF/PSF, central/peripheral skin folds. ^a^ Pearson's correlation test. ^b^ Spearman's correlation test. ^c^ p<0.05. ^d^ p<0.01. ^e^ p<0.001.

Table 5 shows the correlation coefficients among adolescents at risk for overweight/overweight. BMI correlated with all measures of fat distribution, except WTR and CSF/PSF. The BF%, in turn, correlated with all measures of fat distribution, except WHR, CI, SAD, CD and CSF/PSF. Height showed a positive correlation with UC, HC, thigh and SAD. 

**Table 5:** Coefficient of correlation between measures of fat distribution with total body mass, body fat percentage and height in adolescents with excess weight (G3) (n = 35).

Variables	Measures of fat distribution												
	WC	UmC	HC	Thigh	WHR	WHtR	WTR	CI	SAD	CD	CSF	PSF	CSF/PSF
BMI	0.83^b.e^	0.87^b.e^	0.75^b.e^	0.65^b.e^	0.38^b.c^	0.71^b.e^	0.26^b^	0.38^b.c^	0.75^b.e^	0.76^b.e^	0.62^b.e^	0.47^b.d^	0.12^b^
BF%	0.47^b.d^	0.65^b.e^	0.55^b.e^	0.41^b.c^	0.11^b^	0.48^b.d^	0.34^b.c^	0.32^b^	0.28^b^	0.30^b^	0.67^b.e^	0.44^b.c^	0.23^b^
Height	0.30^b^	0.39^a.c^	0.46^a.d^	0.34^b.c^	—0.06^a^	—0.13^b^	0.20^a^	—0.04^a^	0.40^b.c^	0.34^b^	0.04^b^	0.25^b^	—0.30^b^

BMI, body mass index; BF%, body fat percentage; WC, waist circumference; UmC, umbilical circumference; HC, hip circumference; WHR, waist/hip ratio; WHtR, waist/height ratio; WTR, waist/thigh ratio; CI, conicity index; SAD, sagittal abdominal diameter; CD, coronal diameter; CSF, central skinfolds; PSF, peripheral skinfolds; CSF/PSF, central/peripheral skin folds. a Pearson's correlation test. b Spearman's correlation test. c p<0.05. d p<0.01. e p<0.001.

Considering the total sample and the analysis per group, the WC and UC showed the strongest correlations with BMI and BF%, in addition to showing a weak to fair association with height in the total sample and in each group. 

In multiple linear regression analysis between each measure of fat distribution and BF% (dependent variable), after adjustment for age and nutritional status, it was observed that WC, UC, HC, thigh, SAD and CSF showed significant predictive capacity (*p*<0.05) of BF% (Table 6). When the model included all measures of body fat, just UC remained as a significant predictor (*p*=0.038); however, the final model indicated multicollinearity (VIF: 11.18; if VIF < 4 there is no multicollinearity) (data not shown in table).

**Table 6:** Multiple linear regression analysis between the measurements of body fat distribution and BF%, adjusted for age and nutritional status.

Explanatory variables	Coefficients of the independent variables (β)	p	R^2^
WC	0.316	0.008	0.781
UmC	0.390	0.001	0.792
HC	0.446	0.001	0.787
Thigh	0.269	0.021	0.777
WHR	—0.004	0.978	0.766
WHtR	0.196	0.106	0.771
WTR	0.218	0.099	0.772
CI	0.373	0.104	0.771
SAD	0.301	0.011	0.774
CD	0.131	0.376	0.754
CSF	0.120	0.003	0.775
PSF	0.087	0.073	0.762
CSF/PSF	0.089	0.081	0.762

BMI, body mass index; BF%, body fat percentage; WC, waist circumference; UmC, umbilical circumference; HC, hip circumference; WHR, waist/hip ratio; WHtR, waist/height ratio; WTR, waist/thigh ratio; CI, conicity index; SAD, sagittal abdominal diameter; CD, coronal diameter; CSF, central skinfolds; PSF, peripheral skinfolds; CSF/PSF, central/peripheral skin folds.

## Discussion

The present study investigated the correlation between BMI, BF% and height, with measures of body fat content in female adolescents with different levels of adiposity. The results showed that the increase in central fat, represented by WC, UC, WHtR, SAD, CD and CSF, and in peripheral fat, indicated by HC, thigh and PSF was proportional to the increase in BMI and body fat. Vieira et al.^24^ found significantly higher mean values of WC, HC and WHR in normal adolescents with high percentage of body fat when compared to those with normal fat percentage. Similar to the abovementioned study, the results indicate that adolescents with normal weight and excess body fat (G1) had a higher proportion of central fat, represented by the different measures of fat distribution, compared to normal weight ones with adequate body fat (G2); i.e., even though the adolescents were considered as having normal weight according to the BMI, they had excess total body fat and this was reflected in the increase in central fat. These results confirm the limitation of BMI to report on adiposity, particularly on an outpatient basis, and reinforce the importance of routine evaluation of body fat composition and distribution in adolescents. 

In the correlation analysis, it was observed that the WC, UC, WHtR, SAD, CD, CSF, HC, thigh and PSF measures were the ones most associated with BMI and BF%, and WC and UC had the best performance compared to the others. In the group analysis, the largest number of correlations between measures of fat distribution with BMI and BF% was found in the group with excess weight and body fat. A greater proportion of trunk fat with increased BMI has been previously demonstrated in children and adolescents.^12^

Regarding WC, it has been previously shown that it is highly correlated with BMI (r=0.89, *p*=0.001) in female adolescents.^25^ Janssen et al.^26^ also found a similar correlation between BMI and WC (r=0.92 to 0.94) in a study with 2,597 children and adolescents aged 5-18 years. Considering the strong correlation between the two, it may be inferred that such parameters are virtually identical, having no independent effect. However, when evaluating the clinical usefulness of their combined use in a categorized manner, it was observed that the covariance between them is reduced, and thus the combined use of BMI and WC would be a better predictor of health risk for children and adolescents. 

Regarding BF%, a similar study observed a higher correlation between WC and BF% (r=0.85, *p*<0.001) in overweight adolescents (12-18 years) than the present study, when assessed by bipolar electrical bioimpedance, which may have occurred due to possible differences between bipolar and tetrapolar models, in addition to the younger age range evaluated in the aforementioned study, which must have included adolescents whose menarche occurred recently or who had not yet had it, and the associations between abdominal fat and total body fat, which are altered during the sexual maturation process.^25^

Although the WC is a broadly used measure, there is a variety of locations used for its measurement,^27^ and there are no methodological standards, thus making it difficult to compare studies. This study evaluated and compared WC and UC, with close correlations of these measurements being observed with BMI and BF%, except in the group with excess weight, in which associations with UC were stronger.This may reflect a preferential accumulation of fat in the umbilical region rather than in the natural waist, with the increased weight and body fat in adolescents. As it is important to monitor the growth and development of adolescents over time, it is advisable to be consistent and use a single anatomical point for measuring the waist. Considering that the multiple linear regression analysis indicated that the UC was the main predictor of BF%, even after adjusting for age, nutritional status and by other measures of fat distribution, we recommend the use of this standardized anatomical point for waist measurement.

WHR may reflect different aspects of body composition (fat tissue, muscle mass and skeletal structure), and for a given value, there can be large variations in the level of total body fat and visceral adipose tissue.^28^ In the present study, WHR showed a lower correlation than the WC to estimate BF%, but the HC showed a similar correlation to the two anatomical points of waist measurement. Thus, it can be stated that in cases where the WC measurement is extremely difficult to be obtained due to an excessive accumulation of abdominal fat, HC could be a good choice regarding adiposity. 

Oliveira et al^25^ found in females that WHR showed weaker correlations with BMI (r=0.51, *p*=0.03) and BF% (r=0.50, *p*=0.001) than WC, demonstrating that this marker is less dependent on total body fat. Independent effects of waist and hip can be confounded in WHR, indicating that this index has low sensitivity to identify body changes in puberty.^27^

The thigh circumference, similar to HC, also comprises a peripheral measure of fat content. The analyses indicated close correlations between the two measures for both BMI and BF% in eutrophic adolescents and those with excess body fat. As an advantage, unlike HC, the thigh circumference is not affected by variations in the pelvic architecture.^13 ^The weak correlations found for WTR, an index seldom used in adolescents, may be due to the reason mentioned for WHR, i.e., the isolated effect of the measures seems to be diluted when using the ratio between them. Apparently, the use of UC and thigh circumference measures separately, when compared to the use of WTR, has a better performance in predicting adiposity. Recently, it has been proposed the use of the thigh circumference and HC as alternatives to evaluate changes related to growth in body composition and proportions, in places where no imaging methods are available.^28^

One question that has been discussed is whether the use of waist combined with height would be superior to waist circumference alone in predicting cardiovascular risk.^29^ Although the precise effect of height on WC is quantitatively unknown, it is known that it influences the magnitude of WC throughout growth and also in adult life.^4^ In this study, it was observed that the WC and the WHtR showed close correlations with BMI and BF%, except in G2, in which the WHtR, unlike the WC, showed no association with BF%. 

In general, the CI was not a good indicator of body mass and total body fat. In a study with children and adolescents, this index was not a good indicator of fat content in the trunk, probably because the associations between the measures are not good indicators of obesity.^12^ Moreover, the SAD has been reported as similar or even superior to WC as a predictor of metabolic risk in adults.^4^^,^ 5 This study did not assess metabolic parameters, but we observed a similar correlation between these measures with BMI and BF% in the general population. CD had not yet been evaluated in adolescents, and this is the first study about it. In adult women, there was a strong correlation (r=0.91, *p*<0.001) between this diameter and total adipose tissue evaluated by MRI.^20^ In the present study, the CD showed similar behavior to the SAD, demonstrating a relative dependence between height and the abdominal width.

Regarding the skinfolds, in general, the CSF showed a stronger correlation with BMI and BF% than PSF, and both had a higher association than the CSF/PSF ratio. This is probably due to the small variation in ratio values. In the case of overweight adolescents with high body fat, it is particularly important to consider the fragility of the skin folds in predicting body fat, as the thickness of the folds often exceeds the recommended limit (>40 mm) to obtain good quality measurements.^30^

This study also aimed to assess the influence of height on measures of fat distribution. Height was positively correlated with HC, and negatively with WHtR. In the control groups, besides these two, WC, UC, thigh, SAD and CD showed a significant correlation, demonstrating that they seem to be influenced by the adolescents' height. The strongest correlation was with HC, which derives from the fact that this measure is influenced by the skeletal structure.^13^ Its association with WHtR is probably due to the fact that it participates in the ratio, as well as the influence it would have on the WC, as previously discussed. Weaker correlations were observed for WC and UC. It is important to mention that a low correlation with height is desirable for any indicator of obesity. As height increases with age, the strong correlation of an indicator of fat distribution with height may disguise the true association with adiposity.^27^

Several anthropometric indicators of fat distribution have been proposed in the literature as predictors of body fat level and its distribution.^9^^,^ 13 ^,^ 19 ^,^ 25 ^,^ 30 However, such surveys are limited regarding the number of evaluated anthropometric measurements. Dissimilarly, this controlled cross-sectional study was based on the measurement of several circumferences, skinfold thicknesses and diameters; however, one limitation is the lack of data from the early adolescent years, thus restricting the recommendations for the final phase. Considering that anthropometric measures in the assessment of body composition in adolescents have good accuracy,^30^ and that excess body fat, mainly abdominal fat, is related to dyslipidemia, hypertension and insulin resistance as early as in adolescence,^2^^,^ 6 ^,^ 13 the assessment of body fat distribution should be routine in pediatric care. 

It can be concluded that the increase in central and peripheral fat was proportional to the increase in BMI and body fat, indicating collinearity between the specific fat deposits with total fat. The waist and umbilical circumferences were the body fat measure locations that showed the strongest correlations with BMI and BF%, in addition to showing a weak to fair association with height in the total sample and in each group. A weak correlation between anthropometric measures and height is desirable, especially in a period of intense growth, to prevent height from concealing the real association with adiposity. As for the anatomical location of the waist measurement, the umbilicus location was more related to adiposity than the smallest waist point in the overweight group. 

As it is important to monitor the growth and development of adolescents over time, it is advisable to standardize the use of one measure of body fat location. Considering that abdominal fat, more than total fat, has been associated with cardiometabolic risk, it is recommended the use of waist circumference measured at the umbilicus as a measure that reflects the adipose tissue in this region, associated at least with BMI, in the assessment of the nutritional status of adolescents.
