# Supplementary material for: Measurements of body fat distribution: assessment of collinearity with body mass, adiposity and height in female adolescents
Source: Rev Paul Pediatr. 2015 Mar;33(1):63–71. doi: 10.1016/j.rpped.2014.11.011 (PMC4436958; doi:10.1016/j.rpped.2014.11.011)
Supplement: Translation

# Translation: Medidas de localização da gordura corporal: uma avaliação da colinearidade com massa corporal, adiposidade e estatura em adolescentes do sexo feminino

**Authors:** Patrícia Feliciano Pereira, Hiara Miguel Stanciola Serrano, Gisele Queiroz Carvalho, Sônia Machado Rocha Ribeiro, Maria do Carmo Gouveia Peluzio, Sylvia do Carmo Castro Franceschini, Silvia Eloiza Priore

**Affiliations:** Universidade Federal de Viçosa (UFV), Viçosa, MG, Brasil

**Keywords:** Obesidade, Adolescentes, Distribuição de gordura corporal, Antropometria

## Abstract

**OBJETIVO:**

Verificar a correlação entre medidas de localização da gordura corporal com índice de massa corporal (IMC), percentual de gordura corporal (%GC) e estatura, de acordo com o estado nutricional em adolescentes do sexo feminino.

**MÉTODOS:**

Realizou-se estudo transversal controlado, com 113 adolescentes (G1: 38 eutróficas mas com gordura corporal elevada; G2: 40 eutróficas e G3: 35 com excesso de peso), de 14 a 19 anos, de escolas públicas de Viçosa-MG. Aferiu-se peso, estatura, circunferência da cintura (CC), circunferência umbilical (CUm), circunferência do quadril (CQ), circunferência da coxa, relação cintura/quadril (RCQ), relação cintura/estatura (RCE), relação cintura/coxa (RCC), índice de conicidade (IC), diâmetro abdominal sagital (DAS), diâmetro coronal (DC), pregas cutâneas centrais (PCC) e periféricas (PCP). Avaliou-se o %GC por bioimpedância elétrica tetrapolar.

**RESULTADOS:**

O aumento da gordura central, representada pela CC, CUm, RCE, DAS, DC e PCC, e o aumento da gordura periférica indicado pela CQ e da coxa foram proporcionais ao aumento do IMC e %GC. A CC e principalmente CUm apresentaram as correlações mais fortes com a adiposidade, enquanto RCQ, RCC, IC e PCC/PCP as mais fracas. A estatura apresentou correlação com praticamente todas as medidas de localização de gordura, sendo de fraca a regular com as medidas da cintura.

**CONCLUSÕES:**

Os resultados indicam colinearidade entre massa corporal e adiposidade total com tecido adiposo central e periférico. Recomenda-se o emprego da CUm na avaliação do estado nutricional de adolescentes, pois ela apresentou maior capacidade para predizer adiposidade em cada grupo, além de correlação fraca a regular com a estatura.

## Introdução

A adolescência é iniciada com as mudanças corporais da puberdade, sendo um período de grandes modificações psicossociais e físicas. Dentre estas, ressalta-se o intenso crescimento que interfere no acúmulo e na distribuição da gordura corporal.^1^ ^and^ 2 Estudos clínicos e epidemiológicos têm estabelecido que a distribuição da gordura corporal está relacionada a fatores de risco cardiovasculares em adultos^3^ ^and^ 4 e também em crianças e adolescentes.^5^ ^and^ 6 O emprego de medidas válidas na avaliação da composição corporal e do padrão de distribuição de gordura é necessário nos estudos populacionais e na prática clínica, de forma a identificar precocemente indivíduos em risco de desenvolvimento de doenças e auxiliar na prevenção/tratamento da obesidade.^7^

A distribuição de gordura corporal pode ser avaliada por diferentes métodos, como a tomografia computadorizada e a ressonância magnética, equipamentos que apresentam maior precisão e que medem a quantidade de gordura visceral diretamente, mas apresentam custo elevado e necessitam de grande treinamento dos avaliadores, além da tomografia envolver exposição à radiação.^7^ A radioabsorciometria de feixes duplos (DEXA), assim como a antropometria e a bioimpedância elétrica (BIA), não diferencia a gordura subcutânea da visceral. A BIA, embora não seja o método mais preciso para avaliação da composição corporal, é rápido e conveniente para emprego em estudos de campo.^8^ ^and^ 9 As medidas antropométricas incluem perímetros corporais, espessura de dobras cutâneas e alguns diâmetros, que apresentam a vantagem de serem relativamente simples, de baixo custo e inócuas, além de apresentarem bom desempenho na predição da gordura visceral e de risco cardiovascular.^10^ ^and^ 11

Diversas medidas antropométricas de localização da gordura corporal têm sido utilizadas em crianças e adolescentes e não se tem definida qual a melhor medida para uso em pediatria.^2^^,^ 5 ^,^ 6 ^and^ 12 Não está claro se o aumento na adiposidade em crianças e adolescentes se relaciona ao aumento na gordura intra-abdominal.^13^ Diante disso, o presente estudo objetivou verificar a correlação entre medidas de localização de gordura periférica e central propostas na literatura com o IMC, percentual de gordura corporal e estatura, de acordo com o estado nutricional em adolescentes do sexo feminino.

## Método

Realizou-se um estudo transversal com 113 adolescentes do sexo feminino com idades entre 14 e 19 anos de escolas públicas de Viçosa-MG. Para a seleção das participantes, foi realizada uma triagem nas escolas utilizando as medidas de peso e estatura, para determinar o IMC, bem como a medida do %gordura corporal por meio da bioimpedância elétrica bipolar (*Tanita* *(r)*, modelo 2220). As adolescentes também foram questionadas se já haviam apresentado a menarca e data da ocorrência da mesma. As medidas foram obtidas individualmente, em uma sala ou local criado para esse fim, dentro das escolas. As adolescentes que atenderam aos critérios, foram convidadas para uma segunda avaliação realizada no Setor de Nutrição da Divisão de Saúde da Universidade Federal de Viçosa (UFV), onde foram coletadas as medidas antropométricas e de composição coporal. A amostra final foi composta por 38 eutróficas (percentil de IMC entre 5 e 85) 14 mas com elevado percentual de gordura corporal (>28%) (G1-Grupo de estudo), 40 adolescentes eutróficas pelo IMC e com percentual de gordura normal (20-25%) (G2-Grupo controle) e 35 com risco de sobrepeso/sobrepeso classificadas utilizando as curvas do *Center for Disease Control and Prevention* (CDC) (percentil de IMC≥85) 14 e elevado percentual de gordura corporal (>28%) (G3-Grupo controle). As adolescentes incluídas apresentaram menarca há pelo menos 1 ano, o que corresponde a maior chance de terem ultrapassado o período mais intenso de transformações físicas próprias da puberdade. 15 O cálculo amostral foi feito com o programa Epi Info 6.04 para estudos transversais, considerando a população do município de 4.507 indivíduos 16 na faixa etária e sexo do estudo, prevalência de excesso de gordura corporal estimada em 25%, 15 variabilidade de 10% e 95% de intervalo de confiança, resultando tamanho amostral mínimo de 35 indivíduos para cada grupo.

Este trabalho foi aprovado pelo Comitê de Ética e Pesquisa com Seres Humanos da UFV. A participação foi voluntária após esclarecimento verbal e através do termo de consentimento livre e esclarecido, assinado pelos adolescentes e pelos pais e/ou responsáveis.

O peso foi obtido em balança digital eletrônica, com capacidade de 150 kg e divisão de 50 g. A estatura foi aferida utilizando estadiomêtro, com extensão de 2,00 m, dividido em centímetros e subdividido em milímetros. Todas as aferições seguiram as técnicas propostas por Callaway.^17^ O IMC foi calculado através da relação entre peso corporal total (kg) e estatura (m^2^).

O percentual de gordura corporal foi avaliado pela bioimpedância elétrica tetrapolar (*Biodynamics(c), modelo 310, versão 7.1*). A avaliação foi realizada entre 7:00 horas e 8h30 min, com as adolescentes em jejum de 12 horas e seguindo o protocolo específico para este tipo de avaliação. 18

A circunferência abdominal foi aferida em dois locais: menor perímetro do abdômen (circunferência da cintura) e em nível umbilical (circunferência umbilical), sob roupas e no final de uma expiração normal, utilizando uma fita métrica flexível e inelástica.^17^ O quadril foi aferido no maior perímetro da região glútea,^17^ sobre roupas leves. O perímetro da coxa foi medido 3 cm acima da patela no lado esquerdo do corpo em indivíduos cuja mão direita era dominante e no lado direito do corpo naqueles em que a mão esquerda era a dominante.^19^ As medidas foram tomadas duas vezes e utilizou-se a média entre elas. Procedeu-se ao cálculo dos índices RCQ pela razão entre as medidas de cintura e quadril; RCC pelo quociente entre a circunferência umbilical e da coxa; e RCE por meio do quociente entre a circunferência da cintura e a estatura.

O índice de conicidade (IC) foi calculado pela seguinte fórmula:^12^





A distância entre as costas e o abdômen (diâmetro abdominal sagital, DAS) e a distância entre as cristas ilíacas (diâmetro coronal, DC) foram aferidas com a adolescente em posição supina, joelhos inclinados sobre uma superfície plana e firme, sob roupas e após uma expiração normal. Foi marcado o ponto médio entre as cristas ilíacas e depois feita a leitura em nível da crista ilíaca direita, tomando cuidado para não comprimir os tecidos, utilizando um paquímetro de metal com extensão de 50 cm, dividido em centímetro e subdividido em milímetros (Cescorf(r)).^11^ ^and^ 20

As pregas cutâneas subescapular, supra-ilíaca, tricipital e bicipital foram verificadas no lado direito do corpo e todas as medidas foram tomadas por um único avaliador. Cada medida foi verificada três vezes de forma não consecutiva (usou-se a média), utilizando o equipamento *Lange Skinfold Caliper*. 21 Em caso de divergência entre os três valores encontrados superior a 10%, a aferição foi repetida. Considerou-se prega cutânea periférica (PCP) a soma das pregas tricipital e bicipital, e como central (PCC), o somatório da subescapular e suprailíaca, a partir das quais calculou-se a relação PCC/PCP. 22

Na análise estatística, primeiro foi verificada a distribuição das variáveis por meio do teste de normalidade de *Kolmogorov-Smirnov*. A análise exploratória dos dados foi realizada por medidas de tendência central e de dispersão. Posteriormente, para identificar diferença estatística nas variáveis em estudo entre os três grupos de estado nutricional, foi empregado o teste de *Mann-Whitney* e/ou teste *t Student*, de acordo com a distribuição das variáveis. Foi feita correlação de *Pearson* e de *Spearman* entre as variáveis antropométricas e de composição corporal com as medidas de localização de gordura, de acordo com a normalidade das variáveis. A avaliação qualitativa do grau de correlação entre as variáveis seguiu o critério de Callegari-Jacques 23 (correlação nula: r=0; fraca: 0-0,3; regular: 0,3-0,6; forte: 0,6-0,9; muito forte: 0,9-1). As análises foram realizadas no software Sigma-Statistic 2.0 e *STATA*, versão 11.0. A significância estatística considerada foi *p*<0,05.

## Resultados

As características da população estudada encontram-se na tabela 1. A média de idade e de estatura não diferiu entre os grupos, refletindo homogeneidade entre os mesmos. As variáveis peso, IMC, %GC, circunferência da cintura (CC), circunferência umbilical (CUm), quadril, coxa, RCE, DAS, DC, RCC e PCP no grupo G1 foram significantemente maiores que no G2 e menores comparadas ao G3 (*p*<0,001). A RCQ e RCC não diferiram entre G1 e G2. Entre G1 e G3 não se observou diferenças estatisticamente significantes nos índices RCC, IC e PCC/PCP.

**Tabela 1:** Idade, antropometria e composição corporal das adolescentes

	Grupo 1 (n=38)		Grupo 2 (n=40)		Grupo 3 (n=35)	
	±DP	Mediana (faixa)	±DP	Mediana (faixa)	±DP	Mediana (faixa)
Idade (anos)	15,9 (1,3)	15,6 (14-18,8)	15,9 (1,3)	15,8 (14,0-18,0)	15,7 (1,1)	15,4 (14,0-17,9)
Peso (kg)	57,7 (6,3)	57,0 (46,50-75,4)	51,2 (6,0)^ and ^	49,9 (43,0-67,8)	70,0 (12,7)	67,5 (55,4-116,0)^ and ^
Estatura (m)	1,62 (0,06)	1,62 (1,50-1,80)	1,61 (0,07)	1,61 (1,48-1,78)	1,6 (0,06)	1,59 (1,49-1,74)
IMC (kg/m^2^)	21,9 (1,75)	21,7 (19,2-25,2)	19,7 (1,5)	19,4 (17,8-23,4)^ and ^	27,3 (4,03)	25,9 (23,4-41,4)^ and ^
%GC	30,6 (1,8)	30,2 (28,2-35)	22,7 (1,3)	22,9 (20,1-24,7)^ and ^	33,6 (3,3)	32,6 (29,2-42,4)^ and ^
CC (cm)	70,9 (7,9)	69,6 (61,2-111)	65,2 (3,1)	64,7 (60,4-72,7)^ and ^	79,1 (7,8)	76,8 (67,6-105,2)^ and ^
CUm (cm)	79,1 (5,5)	78,9 (69,0-91,9)	72,3 (4,3)	72,3 (64,8-83,7)^ and ^	89,5 (9,4)	88,0 (73,8-88)^ and ^
CQ (cm)	97,8 (5,3)	97,2 (89,0-109,6)	91,8 (4,8)^ and ^	91 (86,0-104,7)	106,7 (8,0)^ and ^	105,3 (94,9-134,8)
Coxa (cm)	40,4 (2,7)	40,7 (34,5-46,0)	37,9 (2,3)^ and ^	37,6 (34,5-43)	44,8 (4,1)	44,4 (38,5-57,9)^ and ^
RCQ	0,73 (0,09)	0,72 (0,62-1,21)	0,71 (0,03)	0,71 (0,60-0,80)	0,74 (0,05)	0,74 (0,66-0,85)^ and ^
RCE	0,44 (0,05)	0,43 (0,37-0,72)	0,41 (0,02)	0,41 (0,36-0,45)^ and ^	0,50 (0,05)	0,48 (0,43-0,63)^ and ^
RCC	1,96 (0,13)	1,95 (1,73-2,25)	1,91 (0,11)	1,91 (1,70-2,10)	2,00 (0,13)	2,00 (1,70-2,30)
IC	1,07 (0,12)	1,06 (0,9-1,7)	1,04 (0,03)^ and ^	1,04 (0,90-1,10)	1,08 (0,04)	1,09 (0,90-1,20)
DAS	17,4 (0,9)	17,3 (15,3-18,9)	15,9 (1,1)^ and ^	15,8 (14,2-18,2)	20,0 (2,3)	19,7 (16,5-26,3)^ and ^
DC	30 (1,7)	30,2 (26,5-32,9)	27,7 (1,6)^ and ^	27,5 (25,0-31,0)	33,4 (2,7)^ and ^	33,8 (28,5-39,2)
PCC	56,5 (12,8)	53,5 (32,0-77,0)	39,2 (7,6)	38,0 (20,0-69,0)^ and ^	72,9 (13,5)^ and ^	72,0 (55,0-114,0)
PCP	38,5 (6,5)	38,5 (27,0-52,0)	30,8 (5,4)	30,0 (23-49,0)^ and ^	49,9 (9,9)^ and ^	49,0 (33,0-72,0)
PCC/PCP	1,47 (0,26)	1,43 (1,00-2,29)	1,28 (0,19)^ and ^	1,29 (0,69-1,57)	1,48 (0,21)	1,50 (1,08-1,78)

IMC, índice de massa corporal; %GC, percentual de gordura total; CC, circunferência da cintura; CUm, circunferência umbilical; CQ, circunferência do quadril; RCQ, relação cintura/quadril; RCE, relação cintura/estatura; RCC, relação cintura/coxa; IC, índice de conicidade; DAS, diâmetro abdominal sagital; DC, diâmetro coronal; PCC, pregas cutâneas centrais; PCP, pregas cutâneas periféricas; PCC/PCP, pregas cutâneas centrais/periféricas. Teste t Student e Teste de Mann-Whitney:

a Diferença entre G1 e G2.

b Diferença entre G1 e G3.

c *p*<0,05.

d *p*<0,001.

A tabela 2 apresenta o coeficiente de correlação entre as variáveis antropométricas e de composição corporal na população total. O IMC e o %GC apresentaram forte correlação com as medidas de localização de gordura corporal, exceto com a RCQ, RCC, IC e PCC/PCP. Para estas, as correlações foram de fraca a regular. As correlações mais fortes foram encontradas entre IMC e CC (r=0,90; *p*<0,001) e entre %GC e CUm (r=0,76; *p*<0,001). A estatura apresentou correlação estatisticamente significante (embora fraca) positiva com CQ e negativa com RCE.

**Tabela 2:** Coeficiente de correlação entre medidas de localização de gordura com massa corporal total, percentual de gordura corporal e estatura na população total (n=113)

Variáveis	Medidas de localização da gordura												
	CC	CUm	CQ	Coxa	RCQ	RCE	RCC	IC	DAS	DC	PCC	PCP	PCC/PCP
IMC	0,90^ and ^	0,89^ and ^	0,89^ and ^	0,88^ and ^	0,28^ and ^	0,89^ and ^	0,25^ and ^	0,34^ and ^	0,86^ and ^	0,83^ and ^	0,82^ and ^	0,77^ and ^	0,41^ and ^
%GC	0,73^ and ^	0,76^ and ^	0,72^ and ^	0,67^ and ^	0,26^ and ^	0,71^ and ^	0,31^ and ^	0,39^ and ^	0,74^ and ^	0,67^ and ^	0,75^ and ^	0,69^ and ^	0,37^ and ^
Estatura	0,14^b^	0,18^b^	0,26^ and ^	0,14^a^	-0,13^a^	-0,26^ and ^	0,09^a^	-0,04^a^	0,11^b^	0,14^b^	-0,009^b^	-0,02^b^	0,01^a^

IMC, índice de massa corporal; %GC, percentual de gordura total; CC, circunferência da cintura; CUm, circunferência umbilical; CQ, circunferência do quadril; RCQ, relação cintura/quadril; RCE, relação cintura/estatura; RCC, relação cintura/coxa; IC, índice de conicidade; DAS, diâmetro abdominal sagital; DC, diâmetro coronal; PCC, pregas cutâneas centrais; PCP, pregas cutâneas periféricas; PCC/PCP, pregas cutâneas centrais/periféricas.

a Teste de correlação de Pearson.

b Teste de correlação de Spearman.

c *p* < 0,01.

d *p* < 0,001.

A tabela 3 apresenta o coeficiente de correlação no grupo de adolescentes eutróficas mas com excesso de gordura corporal. O IMC apresentou correlação significante com CC, CUm, CQ, RCE, DAS, DC, PCC e PCP. O %GC não se correlacionou com nenhuma medida de localização de gordura, enquanto que a estatura se correlacionou, mas em grau regular, com CQ e RCE.

**Tabela 3:** Coeficiente de correlação entre medidas de localização de gordura com massa corporal total, percentual de gordura corporal e estatura nas adolescentes eutróficas com gordura corporal elevada (G1) (n=38)

Variáveis	Medidas de localização da gordura												
	CC	CUm	CQ	Coxa	RCQ	RCE	RCC	IC	DAS	DC	PCC	PCP	PCC/PCP
IMC	0,69^ and ^	0,63^ and ^	0,78 ^ and ^	0,78^ and ^	0,07^b^	0,69^ and ^	-0,05^b^	0,09^b^	0,5^ and ^	0,59^ and ^	0,51^ and ^	0,61^ and ^	0,19^b^
%GC	0,26^b^	0,16^b^	0,05^b^	0,12^b^	0,16^b^	0,26^b^	0,06^b^	0,26^b^	0,14^b^	-0,15^b^	0,12^b^	0,17^b^	-0,03^b^
Estatura	0,06^b^	0,25^a^	0,40^ and ^	0,31^a^	-0,15^b^	-0,45^ and ^	-0,07^a^	-0,08^b^	0,10^a^	0,17^a^	0,07^a^	-0,02^a^	0,10^a^

IMC, índice de massa corporal; %GC, percentual de gordura total; CC, circunferência da cintura; CUm, circunferência umbilical; CQ, circunferência do quadril; RCQ, relação cintura/quadril; RCE, relação cintura/estatura; RCC, relação cintura/coxa; IC, índice de conicidade; DAS, diâmetro abdominal sagital; DC, diâmetro coronal; PCC, pregas cutâneas centrais; PCP, pregas cutâneas periféricas; PCC/PCP, pregas cutâneas centrais/periféricas.

a Teste de correlação de Pearson.

b Teste de correlação de Spearman.

c *p* < 0,05.

d *p* < 0,01.

e *p* < 0,001.

Nas adolescentes eutróficas com gordura corporal adequada, o IMC apresentou correlação estatisticamente significante de regular a forte com praticamente todas as medidas de localização de gordura, exceto com RCQ, RCC, IC e PCP. O %GC apresentou correlação regular com CC, CUm, CQ, coxa e DAS. Com relação às medidas de localização de gordura e a estatura, estas apresentaram correlação regular com CC, CUm, CQ, DAS, DC e RCE. Sendo que com a RCE, a correlação foi negativa (r=-0,44) (tabela 4).

**Tabela 4:** Coeficiente de correlação entre medidas de localização de gordura com massa corporal total, percentual de gordura corporal e estatura nas adolescentes eutróficas (G2) (n=40)

Variáveis	Medidas de localização de gordura												
	CC	CUm	CQ	Coxa	RCQ	RCE	RCC	IC	DAS	DC	PCC	PCP	PCC/PCP
IMC	0,68 ^ and ^	0,67 ^ and ^	0,58 ^ and ^	0,78 ^ and ^	-0,02^b^	0,73 ^ and ^	-0,13^b^	-0,03^b^	0,58^ and ^	0,49^ and ^	0,33^ and ^	0,06^b^	0,34^ and ^
%GC	0,42^ and ^	0,43^ and ^	0,42^ and ^	0,31^ and ^	-0,19^b^	-0,03^b^	0,05^b^	-0,01^b^	0,46^ and ^	0,34^b^	0,18^b^	0,02^b^	0,16^b^
Estatura	0,45^ and ^	0,50^ and ^	0,55^ and ^	0,27^a^	-0,19^a^	-0,44^ and ^	0,22^a^	-0,03^a^	0,36^ and ^	0,44^ and ^	0,06^a^	-0,02^b^	0,14 ^a^

IMC, índice de massa corporal; %GC, percentual de gordura total; CC, circunferência da cintura; CUm, circunferência umbilical; CQ, circunferência do quadril; RCQ, relação cintura/quadril; RCE, relação cintura/estatura; RCC, relação cintura/coxa; IC, índice de conicidade; DAS, diâmetro abdominal sagital; DC, diâmetro coronal; PCC, pregas cutâneas centrais; PCP, pregas cutâneas periféricas; PCC/PCP, pregas cutâneas centrais/periféricas.

a Teste de correlação de Pearson.

b Teste de correlação de Spearman.

c *p*<0,05.

d *p*<0,01.

e *p*<0,001.

Na tabela 5, apresenta-se os coeficientes de correlação nas adolescentes com risco de sobrepeso/sobrepeso. O IMC correlacionou-se com todas as medidas de localização de gordura, exceto RCC e PCC/PCP. O %GC por sua vez correlacionou-se com as medidas de localização de gordura, exceto com RCQ, IC, DAS, DC e PCC/PCP. A estatura apresentou correlação positiva com CUm, CQ, coxa e DAS.

**Tabela 5:** Coeficiente de correlação entre medidas de localização de gordura com massa corporal total, percentual de gordura corporal e estatura nas adolescentes com excesso de peso (G3) (n=35)

Variáveis	Medidas de localização da gordura												
	CC	CUm	CQ	Coxa	RCQ	RCE	RCC	IC	DAS	DC	PCC	PCP	PCC/PCP
IMC	0,83^ and ^	0,87^ and ^	0,75^ and ^	0,65^ and ^	0,38^ and ^	0,71^ and ^	0,26^b^	0,38^ and ^	0,75^ and ^	0,76^ and ^	0,62^ and ^	0,47^ and ^	0,12^b^
%GC	0,47^ and ^	0,65^ and ^	0,55^ and ^	0,41^ and ^	0,11^b^	0,48^ and ^	0,34^ and ^	0,32^b^	0,28^b^	0,30^b^	0,67^ and ^	0,44^ and ^	0,23^b^
Estatura	0,30^b^	0,39^ and ^	0,46^ and ^	0,34^ and ^	-0,06^a^	-0,13^b^	0,20^a^	-0,04^a^	0,40^ and ^	0,34^b^	0,04^b^	0,25^b^	-0,30^b^

IMC, índice de massa corporal; %GC, percentual de gordura total; CC, circunferência da cintura; CUm, circunferência umbilical; CQ, circunferência do quadril; RCQ, relação cintura/quadril; RCE, relação cintura/estatura; RCC, relação cintura/coxa; IC, índice de conicidade; DAS, diâmetro abdominal sagital; DC, diâmetro coronal; PCC, pregas cutâneas centrais; PCP, pregas cutâneas periféricas; PCC/PCP, pregas cutâneas centrais/periféricas.

a Teste de correlação de Pearson.

b Teste de correlação de Spearman.

c *p*<0,05.

d *p*<0,01.

e *p*<0,001.

Considerando a amostra total e a análise por grupo, a CC e CUm apresentaram as correlações mais fortes com IMC e %GC, além de terem apresentado associação fraca a regular com a estatura na amostra total e em cada grupo.

Na análise de regressão linear múltipla entre cada medida de localização de gordura e %GC (variável dependente), após ajuste por idade e estado nutricional, verificou-se que CC, CUm, CQ, coxa, DAS e PCC apresentaram capacidade preditiva significante (*p*<0,05) do %GC (tabela 6). Quando foi feito o modelo incluindo todas as medidas de localização de gordura corporal, apenas a CUm permaneceu como preditor significante (*p*=0,038), contudo o modelo final indicou multicolinearidade (vif: 11,18; se vif <4 não há multicolinearidade) (dados não apresentados em tabela).

**Tabela 6:** Análise de regressão linear múltipla entre as medidas de localização da gordura corporal e %gordura com ajuste por idade e estado nutricional

Variáveis explicativas	Coeficientes das variáveis independentes (ß)	*p*	R^2^
CC	0,316	0,008	0,781
CUm	0,390	0,001	0,792
CQ	0,446	0,001	0,787
Coxa	0,269	0,021	0,777
RCQ	-0,004	0,978	0,766
RCE	0,196	0,106	0,771
RCC	0,218	0,099	0,772
IC	0,373	0,104	0,771
DAS	0,301	0,011	0,774
DC	0,131	0,376	0,754
PCC	0,120	0,003	0,775
PCP	0,087	0,073	0,762
PCC/PCP	0,089	0,081	0,762

Ajuste por idade e estado nutricional. Todas as medidas de localização de gordura foram log transformadas. IMC, índice de massa corporal; %GC, percentual de gordura total; CC, circunferência da cintura; CUm, circunferência umbilical; CQ, circunferência do quadril; RCQ, relação cintura/quadril; RCE, relação cintura/estatura; RCC, relação cintura/coxa; IC, índice de conicidade; DAS, diâmetro abdominal sagital; DC, diâmetro coronal; PCC, pregas cutâneas centrais; PCP, pregas cutâneas periféricas; PCC/PCP, pregas cutâneas centrais/periféricas.

## Discussão

No presente estudo, foi investigada a correlação entre IMC, %GC e estatura com medidas de localização de gordura corporal em adolescentes do sexo feminino com diferentes níveis de adiposidade. Os resultados demonstraram que o aumento da gordura central, representada pela CC, CUm, RCE, DAS, DC e PCC; e da periférica indicada pela CQ, coxa e PCP foi proporcional ao aumento do IMC e da gordura corporal. Vieira et al.^24^ encontraram valores médios de CC, CQ e RCQ significantemente maiores em adolescentes eutróficas com alto percentual de gordura corporal comparada às com percentual normal de gordura. Semelhante ao estudo citado, os resultados indicam que as adolescentes eutróficas e com excesso de gordura corporal (G1) apresentaram maior proporção de gordura central, representada pelas diferentes medidas de localização de gordura, comparada às eutróficas com gordura corporal adequada (G2), ou seja, mesmo as adolescentes estando eutróficas pelo IMC, elas apresentaram excesso de gordura corporal total e este foi refletido no aumento da gordura centralizada. Estes resultados confirmam a limitação do IMC em informar sobre adiposidade, principalmente em nível ambulatorial, e reforçam a importância da avaliação rotineira da composição e distribuição da gordura corporal em adolescentes.

Na análise de correlação, verificou-se que as medidas CC, CUm, RCE, DAS, DC, PCC, PQ, coxa e PCP foram as que mais se associaram ao IMC e %GC, sendo que CC e CUm apresentaram o melhor desempenho, comparado às demais. Na análise por grupo, o maior número de correlações entre as medidas de localização de gordura com IMC e %GC foi encontrada no grupo com excesso de peso e de gordura corporal. Uma maior proporção de gordura no tronco com o aumento do IMC já foi demonstrada anteriormente em crianças e adolescentes.^12^

Em relação à CC, demonstrou-se previamente que esta apresenta alta correlação com IMC (r=0,89; *p*=0,001) em adolescentes do sexo feminino. 25 Janssen et al. 26 também encontraram correlação semelhante entre IMC e CC (r=0,92-0,94) em estudo com 2.597 crianças e adolescentes de 5 a 18 anos. Diante da forte correlação entre ambos, poder-se-ia inferir que tais parâmetros são praticamente idênticos, não tendo efeito independente. Contudo, ao se avaliar a utilidade clínica do uso combinado e de forma categorizada, verificou-se que a covariância entre os mesmos é reduzida e, desta forma, o uso combinado de IMC e CC seria um melhor preditor de riscos à saúde em crianças e adolescentes.

Em relação ao %GC, estudo semelhante observou, em adolescentes (12 a 18 anos) com sobrepeso, maior correlação entre CC e %GC (r=0,85; *p*<0,001) avaliado por bioimpedância elétrica bipolar do que o presente estudo, o que pode ter acontecido pelas possíveis diferenças entre os modelos bipolar e tetrapolar, além da faixa etária mais jovem avaliada no estudo citado, que deve ter incluído adolescentes que ultrapassaram a menarca há pouco tempo ou que ainda não a apresentaram, e as relações entre gordura abdominal com gordura corporal total são alteradas durante o processo de maturação sexual. 25

Embora a CC seja uma medida largamente difundida, existe variedade de locais utilizados na aferição,^27^ não havendo padronização metodológica e dificultando a comparação entre os estudos. Neste trabalho avaliou-se e comparou-se a CC e a CUm, sendo observadas correlações próximas destas medidas com IMC e %GC, exceto no grupo com excesso de peso em que as associações foram mais fortes para CUm. Isto pode refletir um acúmulo preferencial da gordura na região umbilical do que na cintura natural com o aumento do peso e da gordura corporal nas adolescentes. Uma vez que é importante acompanhar o crescimento e o desenvolvimento do adolescente ao longo do tempo, é prudente ser consistente em utilizar um único ponto anatômico para aferição da cintura. Tendo em vista que a análise de regressão linear múltipla indicou que a CUm foi o principal preditor do %GC, mesmo após ajuste por idade, estado nutricional e pelas demais medidas de localização de gordura, recomenda-se o uso padronizado deste ponto anatômico para aferição da cintura.

A RCQ pode refletir diferentes aspectos da composição corporal (tecido adiposo, massa muscular e a estrutura esquelética) e, para um dado valor, pode haver grandes variações no nível de gordura corporal total e de tecido adiposo visceral.^28^ No presente estudo, a RCQ apresentou correlação inferior a CC para estimar %GC, mas a CQ apresentou correlação semelhante aos dois pontos anatômicos de aferição da cintura. Desta forma, pode-se indicar que, para os casos em que a medida de CC é extremamente difícil de ser obtida pelo excessivo acúmulo de gordura abdominal, a CQ poderia ser uma boa opção em relação à adiposidade.

Oliveira et al.^25^ encontraram, no sexo feminino, que a RCQ apresentou correlações mais fracas que a CC com IMC (r=0,51; *p*=0,03) e com o %GC (r=0,50; *p*=0,001), refletindo ser este marcador menos dependente da adiposidade total. Os efeitos independentes da cintura e do quadril podem ser confundidos na RCQ, indicando ter este índice ter baixa sensibilidade para identificar as modificações corporais da puberdade. 27

O perímetro da coxa semelhante à CQ também compreende uma medida periférica de localização da gordura. As análises indicaram correlações próximas entre as duas medidas, tanto com o IMC quanto com o %GC nas adolescentes eutróficas e com excesso de gordura corporal. Como vantagem, ao contrário da CQ, o perímetro da coxa não é afetado por variações na arquitetura pélvica.^13^ As fracas correlações encontradas para a RCC, um índice pouco empregado em adolescentes, podem se dever ao motivo citado para a RCQ, isto é, o efeito isolado das medidas parece ser diluído quando se utiliza a razão entre as mesmas. Ao que tudo indica, o emprego da medidas CUm e perímetro da coxa separadamente, comparado ao uso da RCC, apresenta melhor desempenho para predizer adiposidade. Recentemente tem sido proposto o uso do perímetro da coxa e da CQ como medidas alternativas para avaliar as variações relacionadas ao crescimento na composição corporal e proporções, em locais onde não estão disponíveis métodos de imagem.^28^

Uma questão que vem sendo discutida é se o uso da cintura combinado com a estatura seria superior à circunferência da cintura isolada na predição do risco cardiovascular.^29^ Embora o efeito preciso da estatura sobre a CC seja quantitativamente desconhecido, sabe-se que ela influencia a magnitude da CC ao longo do crescimento e também na vida adulta.^4^ Neste trabalho, observou-se que a CC e a RCE apresentaram correlações próximas com IMC e %GC, exceto no G2 no qual a RCE, ao contrário da CC, não demonstrou associação com o %GC.

De forma geral, o IC não foi bom indicador da massa corporal e da gordura corporal total. Em estudo realizado com crianças e adolescentes, este índice não foi bom indicador da gordura no tronco, provavelmente pelo fato das relações entre medidas não serem bons indicadores da obesidade.^12^ Por outro lado, o DAS tem sido colocado como semelhante ou até superior a CC como preditor de risco metabólico em adultos.^4^ ^and^ 5 Neste estudo, não foram avaliados parâmetros metabólicos, mas observou-se correlação semelhante entre tais medidas com o IMC e o %GC na população geral. O DC ainda não havia sido avaliado em adolescentes, sendo este o primeiro estudo a respeito. Em mulheres adultas, houve forte correlação (r=0,91; *p*<0,001) entre este diâmetro com o tecido adiposo total avaliados por ressonância magnética. 20 No presente estudo, o DC apresentou comportamento semelhante ao DAS, demonstrando uma relativa dependência entre a altura e a largura do abdômen.

Com relação às pregas cutâneas, de forma geral, a PCC apresentou uma correlação mais forte com IMC e %GC do que a PCP, sendo que ambas tiveram maior associação que a relação PCC/PCP. Isto ocorre, provavelmente, devido à pequena variação nos valores da razão. No caso das adolescentes com excesso de peso e gordura corporal elevada, é particularmente importante considerar a fragilidade das pregas cutâneas em predizer a gordura corporal, visto que a espessura das dobras frequentemente ultrapassa o limite recomendável (>40 mm) para obter medidas de boa qualidade.^30^

Neste estudo, também foi objetivo verificar a influência da estatura sobre as medidas de localização de gordura. A estatura apresentou correlação positiva com CQ e negativa com RCE. Nos grupos controles, além destas duas, a CC, CUm, coxa, DAS e DC mostraram correlação significante, demonstrando que elas parecem ser influenciadas pelo valor de estatura das adolescentes. A correlação mais forte foi com a CQ, o que decorre do fato desta medida ser influenciada pela estrutura esquelética.^13^ A associação com a RCE se deve provavelmente ao fato dela participar da relação, bem como pela influência que a mesma teria sobre a CC, como já discutido. As correlações mais fracas foram observadas para a CC e CUm. É importante colocar que uma baixa correlação com a estatura é desejável para qualquer indicador de obesidade. Como a estatura aumenta com a idade, a correlação forte de um indicador de localização de gordura com a estatura pode mascarar a verdadeira relação com a adiposidade.^27^

Diversos indicadores antropométricos de localização de gordura têm sido propostos na literatura como preditores do nível de gordura corporal e da sua distribuição.^9^^,^ 13 ^,^ 19 ^,^ 25 ^and^ 30 Contudo, tais pesquisas são limitadas quanto ao número de medidas antropométricas avaliadas. Ao contrário, este estudo transversal controlado baseou-se na aferição de vários perímetros, pregas cutâneas e diâmetros, entretanto, uma limitação é a falta de dados dos primeiros anos da adolescência, restringindo as recomendações para a fase final. Tendo em vista que as medidas antropométricas na avaliação de composição corporal em adolescentes apresentam boa acurácia^30^ e que o excesso de gordura corporal, principalmente, a gordura abdominal está relacionada a dislipidemias, hipertensão arterial e resistência à insulina já na adolescência,^2^^,^ 6 ^and^ 13 a avaliação da distribuição da gordura corporal deveria ser rotina em pediatria.

Pode-se concluir que o aumento da gordura central e da periférica foi proporcional ao aumento do IMC e da gordura corporal, indicando colinearidade entre os depósitos específicos de gordura com a adiposidade total. As circunferências da cintura e umbilical foram as medidas de localização da gordura corporal que apresentaram as correlações mais fortes com IMC e %GC, além de terem apresentado associação fraca a regular com a estatura na amostra total e em cada grupo. Uma correlação fraca entre uma medida antropométrica e estatura é desejável, principalmente em uma fase de intenso crescimento, para evitar que a estatura mascare a real associação com a adiposidade. Quanto ao local anatômico de aferição da cintura, o ponto na incisura umbilical foi mais relacionado à adiposidade do que o ponto da menor cintura no grupo com excesso de peso.

Uma vez que é importante acompanhar o crescimento e o desenvolvimento do adolescente ao longo do tempo, é prudente padronizar o uso de uma mesma medida de localização da gordura. Tendo em vista que a gordura abdominal, mais que a adiposidade total, tem sido associada ao risco cardiometabólico, recomenda-se o uso da circunferência da cintura aferida na incisura umbilical como uma medida que reflita o tecido adiposo desta região associada pelo menos ao IMC na avaliação do estado nutricional de adolescentes.
